# The Potential of Small Molecules to Modulate the Mitochondria–Endoplasmic Reticulum Interplay in Alzheimer’s Disease

**DOI:** 10.3389/fcell.2022.920228

**Published:** 2022-08-26

**Authors:** Giacomo Dentoni, Laura Castro-Aldrete, Luana Naia, Maria Ankarcrona

**Affiliations:** Division of Neurogeriatrics, Center for Alzheimer Research, Department of Neurobiology, Care Science and Society (NVS), Karolinska Institutet, Stockholm, Sweden

**Keywords:** Alzheimer’s disease, mitochondria–endoplasmic reticulum contact sites, small molecules, neurodegeneration, mitochondrial function

## Abstract

Alzheimer’s disease (AD) is the most common neurodegenerative disease affecting a growing number of elderly individuals. No disease-modifying drugs have yet been identified despite over 30 years of research on the topic, showing the need for further research on this multifactorial disease. In addition to the accumulation of amyloid β-peptide (Aβ) and hyperphosphorylated tau (p-tau), several other alterations have been associated with AD such as calcium (Ca^2+^) signaling, glucose-, fatty acid-, cholesterol-, and phospholipid metabolism, inflammation, and mitochondrial dysfunction. Interestingly, all these processes have been associated with the mitochondria–endoplasmic reticulum (ER) contact site (MERCS) signaling hub. We and others have hypothesized that the dysregulated MERCS function may be one of the main pathogenic pathways driving AD pathology. Due to the variety of biological processes overseen at the MERCS, we believe that they constitute unique therapeutic targets to boost the neuronal function and recover neuronal homeostasis. Thus, developing molecules with the capacity to correct and/or modulate the MERCS interplay can unleash unique therapeutic opportunities for AD. The potential pharmacological intervention using MERCS modulators in different models of AD is currently under investigation. Here, we survey small molecules with the potential to modulate MERCS structures and functions and restore neuronal homeostasis in AD. We will focus on recently reported examples and provide an overview of the current challenges and future perspectives to develop MERCS modulators in the context of translational research.

## 1 Background

Alzheimer’s disease (AD) is a neurodegenerative disorder primarily associated with dementia and pathological features such as extensive neuronal and synaptic loss, accumulation of plaques consisting of amyloid β-peptide (Aβ), and intracellular neurofibrillary tangles (NFTs) formed by hyperphosphorylated tau (p-tau) protein (Braak and Braak, 1991). AD is classified as familial AD (fAD) and sporadic AD (sAD). Most AD cases are sporadic and still lack a clear genetic or environmental component. fAD accounts for less than 1% of diagnosed cases and is caused by the inheritance of autosomal mutations in three genes: amyloid precursor protein (APP), and presenilin (PS) 1 and 2. The amyloid hypothesis has been dominating the field, which assumes that increased proteolytic cleavage of APP and decreased clearance of Aβ_,_ result in several downstream effects leading to tau phosphorylation and aggregation, synapse dysfunction, and neuronal death. However, this theory fails to explain several features of the disorder, thoroughly reviewed in Morris et al. (2014). When the role of Aβ on neurotoxicity is undeniable, AD research is shifting toward considering the disorder as a multifactorial disease, characterized by different types of microdamage and different factors that lead to cognitive decline (Sasaguri et al., 2017).

The drug development pipeline to find a cure or treatment for AD has continuously been challenged and has focused mainly on the amyloid deposition aspects of the disease. Recent evidence suggests that before cognitive decline commences, Aβ accumulation initiates, peaks, and decelerates. Therefore, by the time cognitive impairment is clinically observed, Aβ accumulation has already plateaued (Hardy and Selkoe, 2002; Jack et al., 2010; Karran et al., 2011; Selkoe and Hardy, 2016). Indeed, compounds (e.g., bapineuzumab, solanezumab, and gantenerumab) used in clinical trials to either reduce Aβ_42_ production (e.g., ClinicalTrials.gov identifiers: NCT00470418, NCT01303744, NCT01421056, and NCT00606164) or prevent Aβ aggregation (e.g., NCT00568776 and NCT00934050) have been unsuccessful in slowing down or preventing the pathophysiology of AD (Aisen et al., 2011; Mehta et al., 2017). Although recently the FDA approved immunoglobulin gamma 1 (IgG1) monoclonal antibody aducanumab (brand name Aduhelm) for treatment of certain subgroups of AD patients, a comprehensive analysis of these results is still pending to fully access its neuroprotective effects. The European Medicines Agency (EMA) has recommended that Aduhelm be denied marketing authorization due to its uncertain therapeutic potential and safety concerns (Mahase, 2021). Failures in AD drug development so far might be related to 1) drugs being tested mainly in the late symptomatic stages of AD once irreversible damage has occurred, 2) drugs target advanced stages of disease pathways rather than early modifiable ones, 3) overreliance on the drug development pipeline on fAD mechanisms and amyloid hypothesis, and 4) lack of drive to explore other pathological targets.

Among these underexplored pathological targets in AD drug discovery, reduced glucose metabolism has been reported decades before clinical symptoms manifested (Minoshima et al., 1997; Mosconi, 2005), and multiple studies have revealed mitochondrial dysfunction as an upstream event of Aβ accumulation (Reddy et al., 2004; Lee et al., 2006; Swerdlow, 2012). Indeed, electron transport chain (ETC) inhibition results in upregulated tau phosphorylation and amylogenic pathway activation (Lovell et al., 2004; Leuner et al., 2012; Esteras et al., 2017). Furthermore, introducing mitochondrial DNA of sAD subjects in mitochondrial DNA-depleted cells increases apoptotic mechanisms such as cytochrome *c* release and caspase-3 activation (Khan et al., 2000), abnormal intracellular Ca^2+^ signaling (Sheehan et al., 1997), and oversecretion of Aβ peptides (Khan et al., 2000). Furthermore, decreased mitochondrial protein expression of ETC complexes in the absence of Aβ and tau pathology has been reported in sAD human iPSC-derived neuronal cell models (Birnbaum et al., 2018). Altogether, in the context of clinical trials, these studies suggest that metabolic dysfunctions may precede amyloid pathology, and early treatment in the prodromal stage may increase the likelihood of slowing down AD progression. Drug delivery strategies that work directly or indirectly on mitochondria have been established and studied in neurodegenerative disorders (Cenini and Voos, 2019). Several classes of drugs, with different modes of action, have been tested to tackle mitochondrial dysfunction in AD including antioxidants, compounds targeting mitochondria membrane potential (ΔΨm), and inhibiting mitochondria permeability transition pore (mPTP) opening, as well as natural compounds from the flavonoid family. When mitotherapeutics are gaining momentum, only 10 phase III clinical trials incorporating new drugs for the treatment of mitochondrial illnesses are currently registered, and only one of these 10 trials has been completed (Weissig, 2020). Regarding AD, currently, 12% of drugs under clinical trials in phase III have been shown to impact on metabolism and bioenergetics, showing the promising potential of targeting mitochondria in AD (Cummings et al., 2021).

The cellular pathways regulating mitochondrial function and neuropathological AD-related processes converge at mitochondria–endoplasmic reticulum (ER) contact sites (MERCS), intracellular microdomains which have been receiving increased attention from the scientific community. We and others have hypothesized that upregulated MERCS structures and functions may be one of the main events driving AD pathology (Hedskog et al., 2013; Area-Gomez and Schon, 2017), and it is interesting to note that many dysfunctional mechanisms observed in AD are mediated at this signaling platform. In this review, we summarize up-to-date knowledge on MERCS physiology and its alterations in AD, provide a survey of small molecules with the capacity to modulate MERCS structures and functions to restore neuronal homeostasis, and address new directions for the development of small molecules targeting MERCS. We focus on recently reported cases of pharmaceutical agents proven to influence MERCS structures or functions and provide an overview of the current challenges and future perspectives to develop MERCS modulators in the context of translational research.

## 2 Mitochondria–Endoplasmic Reticulum Contact Sites

### 2.1 Contactology: a New Field for Organelle Interaction

The interaction of different intracellular organelles has been established as a fascinating new subject in cellular biology, and a growing number of cellular physiological and pathological mechanisms have been attributed to these contacts. Csordás, Weaver, and Hajnóczky introduced the name “contactology” to describe this new field of study (Csordás et al., 2018). Membrane contact sites are intracellular regions where the membranes of two organelles are closely juxtaposed. There are some characteristics that all contact sites present including tethering regulators of proximity between two membranes, a distinct lipidome/proteome from the rest of the membranes, and no fusion events between the two membranes (Scorrano et al., 2019). Contacts between different organelles have been described; nevertheless, all best characterized organelle contacts involve interaction with the ER. Contacts between the ER and the plasma membrane, the Golgi apparatus, the vacuole/lysosome, and mitochondria mediate Ca^2+^ homeostasis and lipid shuttling. Additionally, ER interactions with the vacuole/lysosome and mitochondria have been shown to mediate microautophagy and autophagosome formation, respectively (Holthuis and Ungermann, 2013).

### 2.2 Mitochondria–Endoplasmic Reticulum Contact Sites

Mitochondria need to communicate with other organelles, particularly the ER, in order to regulate their functions. Mitochondria-associated ER membranes (MAMs) are lipid raft-like domains in the ER that can interact with OMM. The MAM is an insoluble lipid raft that differs biochemically from the pure ER and pure mitochondria. MERCS are formed when approximately 20% of the mitochondrial surface is closely apposed to MAM (10 to 30 nm distance) (Csordás and Hajnóczky, 2001; Csordás et al., 2006; Paillusson et al., 2016). MERCS were first observed on electron micrographs of the rat liver in the 1950s (Bernhard and Rouiller, 1956). These subcellular regions drew additional attention when Jean Vance identified subcellular fractions enriched in the MAM in the early 1990s, then known as fraction X (Vance, 1990). Since then, the contactology field has advanced significantly, and considerable efforts have been made by the scientific community to characterize the mechanisms that connect the ER to mitochondria.

### 2.3 Linking the Two Membranes: Mitochondria–Endoplasmic Reticulum Contact Site Tethers and Contact Regulators

Generally, 10–30 nm distance between the ER and mitochondria allows for protein-mediated tethers to be formed. The presence of protein bridges between the two structures has been confirmed by the ability of proteolytic agents to untether the two organelles (Csordás et al., 2006). In yeast, the tethering structure linking the two membranes has been thoroughly characterized by containing OMM proteins Mdm34 and Mdm10, cytosolic protein Mdm12, and ER transmembrane protein Mmm1 forming together as the ER–mitochondria encounter structure (ERMES) complex (Puri et al., 2019). So far, no ERMES homologs in mammals have been identified. The mammalian proteins involved in MERCS juxtaposition have been identified upon manipulation of the examined proteins by analyzing proximity and MERCS functional alteration, such as Ca^2+^ shuttling between the ER and mitochondria (Herrera-Cruz and Simmen, 2017). The mammalian proteins that have thus far been discovered are summarized in this study.

Phosphofurin acid cluster sorting protein 2 (PACS2) was identified as the first mediator of the ER to mitochondria juxtaposition in mammalian cells. Considering PACS2 is a cytoplasmic protein rather than a MERCS tether; it may indirectly couple the ER to the mitochondria through upstream pathways. Nonetheless, knocking down this protein reduces MERCS and Ca^2+^ shuttling from the ER to the mitochondria and has been used in earlier studies in the field to modulate MERCS structures and functions (Simmen et al., 2005).

Homodimeric and heterodimeric interactions between mitofusin 1 (Mfn1) and mitofusin 2 (Mfn2), proteins involved in mitochondrial fusion, were first described as a scaffolding bridge between the ER and mitochondria (De Brito and Scorrano, 2008). Studies have shown that deleting the *Mfn2* gene or conditionally knocking down Mfn2 reduces the mitochondria–ER interaction in a variety of models including in cell lines (Alford et al., 2012; Sugiura et al., 2013), neurons (Schneeberger et al., 2013), and astrocytes *in vivo* (Gӧbel et al., 2020). Several investigations, however, have suggested that Mfn2 knockdown promotes MERCS apposition and Ca^2+^ shuttling, implying that this protein acts as a negative regulator of contact formation (Cosson et al., 2012; Filadi et al., 2015; Leal et al., 2016; Cieri et al., 2018a; Dentoni et al., 2022). These differences may be attributed to different cell models and culturing methods employed, variable compensatory mechanisms from Mfn2 knockout or knockdown, and methods used to measure the ER to mitochondria proximity, which have been thoroughly reviewed by Filadi et al. (2018a). Regardless of its role, Mfn2 manipulation has been used and characterized as a method to modify ER–mitochondria proximity.

Another pair of proteins working as *bona fide* tethers are the OMM protein tyrosine–phosphatase-interacting protein 51 (PTPIP51) and the ER protein vesicle-associated membrane protein-associated protein B (VAPB). Indeed, knocking down either protein reduces apposition between the two organelles, while overexpression has the opposite effect (De Vos et al., 2012; Stoica et al., 2014). In particular, knocking down either protein prevents the ER to mitochondria Ca^2+^ shuttling, whereas overexpression increases the functional coupling between the two organelles (Stoica et al., 2014; Puri et al., 2019).

PDZD8, an ER transmembrane protein, has been identified through a modeling screening approach, whereby the synaptotagmin-like mitochondrial-lipid-binding (SMP) domain homology between ERMES proteins and mammalian proteins was assessed. Indeed, the PDZD8 SMP domain shows substantial homology to the ERMES ER protein Mmm1. PDZD8 knockout or knockdown revealed substantial downregulation of MERCS apposition and Ca^2+^ shuttling in cell lines and neurons, shaping dendritic Ca^2+^ fluxes. PDZD8 mitochondrial tethering counterpart remains undetected (Hirabayashi et al., 2017).

The IP3R-Grp75-VDAC bridge is a complex constituted by the inositol 1,4,5-triphosphate receptor (IP3R), glucose-regulated protein 75 (Grp75), and VDAC (voltage-dependent anion channel). Grp75 allows for close apposition of IP3R, a key ER Ca^2+^ release channel, to the OMM and VDAC (Szabadkai et al., 2006), hence constituting the main route of Ca^2+^ transfer from the ER to mitochondria. Previous research has shown that deleting IP3Rs has no effect on the distance between the two organelles (Csordas et al., 2006); this suggests that their apposition to VDAC is purely functional in mediating Ca^2+^ transfer. However, in recent investigations on IP3R-deficient (triple knockout (KO)) DT40 cells, where individual IP3R isoforms were recovered, each IP3R isoform restored Ca^2+^ shuttling and ER–mitochondrial proximity in triple KO cells, indicating a structural involvement in MERCS juxtaposition (Bartok et al., 2019).

Other less studied tethering proteins have been identified in mammalian cells, including B-cell receptor-associated protein 31 (BAP31) and Fis1 (Iwasawa et al., 2011), FATE1 (Doghman-Bouguerra et al., 2016), transglutaminase type 2 (TG2) (D’Eletto et al., 2018), and FK506-binding protein 8 (FKBP8) (Kwak et al., 2020).

Although several approaches have been employed to evaluate tethering mechanisms regulating the ER to mitochondria juxtaposition, only a few studies have looked directly at the proteome of these structures. To date, three proteomic investigations on mouse brain fractions enriched in the MERCS have been conducted, and between 1000 and 2400 proteins have been discovered (Poston et al., 2013; Ma et al., 2017; Wang et al., 2018). A comparison of previously described datasets identified 648 common proteins present in mouse brain MERCS-enriched fractions, including MERCS functional and structural tethering proteins such as PACS, Mfn2, VAPB, IP3R1, and all VDAC isoforms (Magalhães Rebelo et al., 2020). Interestingly, when comparing the proteome of the MERCS-enriched fraction from the brain to the proteome from other tissues and cells, such as the testis, liver, and NG108-15 cell line, only 18 proteins were shown to be common within these databases (Magalhães Rebelo et al., 2020), suggesting a tissue-specific enrichment of proteins at the MERCS (Leal and Martins, 2021).

### 2.4 Mitochondria–Endoplasmic Reticulum Contact Sites’ Functions: a Physiological Subcellular Hub

**FIGURE 1:**
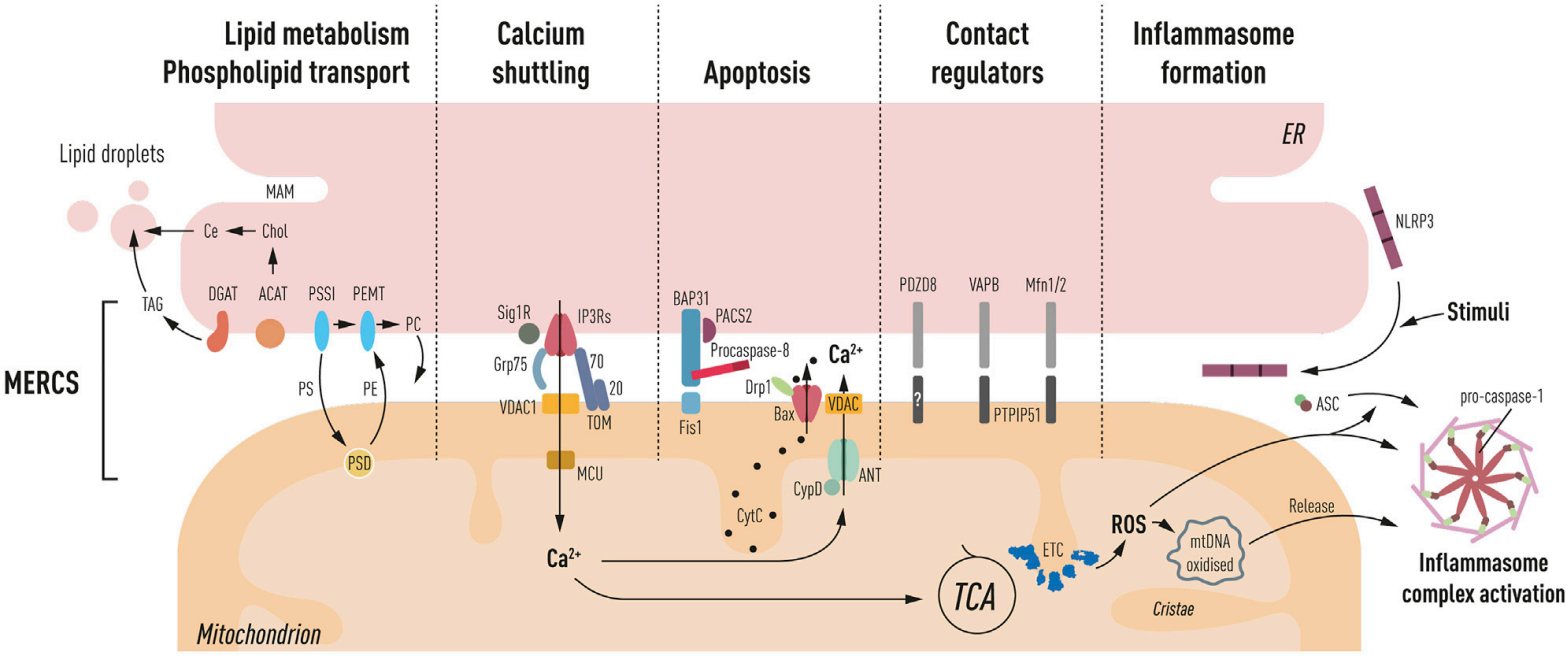
Schematic representation of processes ascribed at MERCS: **lipid metabolism and phospholipid transport**. PS, phosphatidylserine; PE, phosphatidylethanolamine; PC, phosphatidylcholine; shuttling occurs through MAM, OMM, and IMM triple contact sites, and PSSI, PSD, and PEMT mediate their synthesis, respectively. Furthermore, the synthesis of cholesteryl esters (Ce) through ACAT and triglycerides (TAG) through DGAT occurs at the MAM, which is the main component of lipid droplets, generated close to the ER and mitochondria interface. **Ca^2+^ shuttling** between the ER and mitochondria is mediated by IP3Rs. Grp75 mediating close juxtaposition of IP3Rs to VDAC in the OMM and MCU in IMM facilitates Ca^2+^ entry into the matrix leading to TCA cycle activation. Sig1R and TOM70 mediate the IP3R activity. **Apoptosis** PACS-2, a multifunctional sorting protein, by localizing to the MAM regulates BAP31 interaction with Fis1, mediating a platform for procaspase-8 activation. Increased Ca^2+^ in the matrix can lead to activation of mPTP (CyD, ANT, and VDAC). Bax has also been located at MERCS, and upon apoptosis induction, it leads to the formation of pores and cytochrome *c* (CytC) release. **Contact regulators’** several proteins have been shown to have a role in mediating ER to mitochondria apposition including PDZD8, VAPB–PTPIP51, and Mfn2, a negative regulator of MERCS. **Inflammasome formation** NLRP3 under resting conditions is located in the ER membrane, upon inflammatory stimuli recruits ASC and redistributes at the MAM. Mitochondrial ROS production and oxidized DNA release induce the activation of the autophagosome complex, consisting of NLRP3, ASC, and pro-caspase-1 propagating the inflammatory response.

Several proteins have been identified at MERCS acting as functional rather than structural mediators of juxtaposition between the two membranes. These proteins play diverse physiological roles in this subcellular domain (Figure 1).

#### 2.4.1 Endoplasmic Reticulum to Mitochondria Ca^2+^ Shuttling

MERCS play an important role in the ER to mitochondria Ca^2+^ shuttling, generating high Ca^2+^ hotspots (Ca^2+^>10 µM) at the interface between the two membranes, allowing the activation of low-affinity calcium transporters MCU while avoiding an overall cellular rise in Ca^2+^ (Rizzuto et al., 1993). Mitochondrial Ca^2+^ import is carried out through the IP3R-Grp75-VDAC bridge and plays a central bioenergetic function by activating tricarboxylic acid (TCA) cycle dehydrogenases and boosting ATP production (Jouaville et al., 1999; Cardenas et al., 2010a) (depicted in Figure 1), which seems to be the preferential method for boosting metabolism compared to the cytosolic Ca^2+^ influx (Rohács et al., 1997). The ryanodine receptor (RyR) and IP3R are the main Ca^2+^ extrusion channels in the ER. RyR has also been found at MERCS and, like IP3R, it regulates Ca^2+^ shuttling to mitochondria; however, most research on RyR has been conducted in muscle cells (Csordás and Hajnóczky, 2001). This ion’s under- or over-shuttling, as well as the activation of Ca^2+^ channels, can have negative consequences for cellular physiology. Reduced Ca^2+^ shuttling can induce a bioenergetic crisis, whereas pathologically elevated Ca^2+^ levels can promote mitophagy and death (Rowland and Voeltz, 2012). Ca^2+^ responses can be modulated by several proteins in this subcellular area. MERCS-resident protein sigma-1 receptor (Sig1R) modulates the IP3R activity through ankyrin 2 (Ank2) interaction, stabilizing IP3Rs during conditions of high calcium transfer (Hayashi and Su, 2007). Importantly, in Sig1R knockout motor neurons, there is a significant reduction of connections between the two organelles, and whether this is dependent on its interaction with IP3Rs remains unknown (Bernard-Marissal et al., 2015). Upon ER Ca^2+^ depletion and ER stress, Sig1R increases Ca^2+^ shuttling to mitochondria resulting in enhanced ATP (Hayashi and Su, 2007). Furthermore, we discovered that TOM70 operates as an ER–Ca^2+^ regulator by interacting with IP3R3, facilitating Ca^2+^ shuttling at MERCS, and influencing bioenergetics, cell proliferation, and autophagy (Filadi et al., 2018c). Other proteins involved in the stabilization of the IP3R-Grp75-VDAC bridge include Tespa-1 (Matsuzaki et al., 2013), transglutaminase type 2 (TGM2) (D’Eletto et al., 2018), and DJ-1 (Liu et al., 2019). The sacro/endoplasmic reticulum (SR/ER) Ca^2+^ ATPase (SERCA), which replenishes intracellular ER Ca^2+^ stores, has also been shown to control ER–mitochondria Ca^2+^ transfer by attenuating mitochondrial Ca^2+^ uptake during continuous Ca^2+^ release at MERCS (Csordás and Hajnóczky, 2001). ER Ca^2+^ modulators found at MERCS controlling the SERCA activity include calnexin (CNX) (Roderick et al., 2000), calreticulin (John et al., 1998), ERp57 (Li and Camacho, 2004), and the thioredoxin-related transmembrane protein (TMX1) (Raturi et al., 2016).

#### 2.4.2 Phospholipid Shuttling, Cholesterol, and Lipid Metabolism

MAM is a lipid raft domain enriched in cholesterol and sphingolipids, which facilitates the stabilization of specific proteins enriched in these membranes (Vance, 2014). MERCS also play a role in the phospholipid inter-organelle transport by enabling their exchange between the two organelles (Vance, 1990). Indeed, phosphatidylserine (PS) is synthesized at the MAM and then transported to the mitochondria OMM where it is converted into phosphatidylethanolamine (PE) (Vance, 1990) through a triple contact site formed with the ER, outer mitochondrial membrane (OMM), and inner mitochondrial membrane (IMM) (Ardail et al., 1993). PE can remain in the IMM or be transported back to the ER (Vance, 1991). Next, PE can be further modified in the ER to phosphatidylcholine (PC) (depicted in Figure 1). Furthermore, MERCS play a vital role in the metabolism of other lipids, including cholesterol and triglycerides (Vance, 2014). The major components of lipid droplets are triglycerides and sterol esters (Guo et al., 2009), which have been demonstrated to form near the ER–mitochondria contact interface (Perreault et al., 2009). Accordingly, lipid droplet formation has been used as an indirect way to assess phospholipid-related MERCS functions (Area-Gomez et al., 2012; Del Prete et al., 2017). Recently, MERCS tethering protein PTPIP51 has been shown to work as a phospholipid transfer protein. PTPIP51 depletion reduced levels of mitochondrial cardiolipin in cells, a mitochondrial lipid almost exclusively found within the IMM (Yeo et al., 2021).

#### 2.4.3 Initiation of Autophagosome Formation

Autophagosome membrane formation has been identified as another physiological function attributed to MERCS (Hamasaki et al., 2013; Garofalo et al., 2016). MERCS tethering protein VAPB or PTPIP51 overexpression increases ER–mitochondria contacts, impairing autophagosome formation, while downregulation of these proteins decreases contacts, upregulating autophagosome formation (Gomez-Suaga et al., 2017). It is interesting to note that in MERCS where autophagosomes are generated, the distance between the two membranes is further spaced out (50 nm), compared with MERCS mediating Ca^2+^ and lipid transfer (10–30 nm), to adapt to the growing autophagosome membrane formation (Giacomello and Pellegrini, 2016). Thus, MERCS appears to be an active membrane assembly area due to its phospholipid exchange and synthesis of autophagosome membranes.

#### 2.4.4 Apoptosis and Mitochondrial Fission

Although increased apposition between the two organelles is fundamental for bioenergetics, sustained increased connectivity makes mitochondria prone to Ca^2+^ overloading, ensuing mPTP activation (Csordas et al., 2006). Furthermore, ER tethering protein BAP31 interacts with its mitochondrial partner Fis1, which together create a platform for the activation of procaspase 8 (Iwasawa et al., 2011); accordingly, depletion of Fis1 has been shown to delay apoptosis (Lee et al., 2004).

Drp1 mediates mitochondrial division by being recruited at MERCS (Friedman et al., 2011a) *via* the ER-resident inverted formin 2 (INF2) (Korobova et al., 2013). This process requires polymerization of actin near constriction sites and the ER to mitochondria Ca^2+^ shuttling (Chakrabarti et al., 2017). Interestingly, Drp1 is recruited to the mitochondria by the apoptotic mediator Bax during apoptosis at MERCS (Wasiak et al., 2007). Bax and Drp1 colocalization at MERCS coordinate the activation of mitochondria outer membrane permeabilization (MOMP), hence mechanistically linking mitochondria fragmentation to apoptosis.

#### 2.4.5 ROS Production and Inflammasome Formation

Communication between the ER and mitochondria is also important for redox signaling between the two organelles. Ca^2+^ shuttling at MERCS can boost mitochondrial-derived ROS generation, resulting in the generation of MERCS ROS nanodomains (Booth et al., 2016). Indeed, ROS can influence the activity of ER Ca^2+^ channels such as RyR and IP3Rs (Bogeski et al., 2011), thus establishing a bidirectional communication at MERCS.

Moreover, ROS production at MERCS has been shown to induce the assembly and activation of the inflammasome (Zhou et al., 2011), a protein complex formed by nucleotide-binding oligomerization domain (NOD)-like receptor protein 3 (NLRP3), adapter protein apoptosis-associated speck-like protein containing a CARD (ASC), and caspase-1 which results in cleavage and secretion of cytokines, propagating the inflammatory response (Yabal et al., 2019). NLRP3 is activated by a variety of stimuli including ion flow disruption across the plasma membrane, mitochondrial ROS and oxidized mitochondrial DNA (mtDNA) release, lysosomal membrane disruption, and bacterial or viral infection (Missiroli et al., 2018). Interestingly, NLRP3 and ASC have been shown to translocate to MERCS upon triggering stimuli, activating caspase-1 (Zhou et al., 2011; Namgaladze et al., 2019) (depicted in Figure 1). Furthermore, evidence points to Ca^2+^ shuttling between the ER and mitochondria being important for the activation of inflammasomes (Murakami et al., 2012). IP3R inhibition resulted in decreased ROS, mtDNA release, and secretion of cytokines (Triantafilou et al., 2013). From the aforementioned evidence, the NLRP3 inflammasome’s presence at MERCS is strategically placed to detect stress-related mitochondrial signals. The mechanism underlying the ER to mitochondria apposition influencing inflammasome formation and activation remains unexplored.

## 3 Mitochondria–Endoplasmic Reticulum Contact Sites and Neurodegeneration

MERCS are involved in a wide range of cellular mechanisms, as mentioned in the previous section. It is not surprising, then, that MERCS dysfunction has been linked to several neurodegenerative disorders. Although it is uncertain whether MERCS play an active function in disease development, numerous pieces of evidence suggest that MERCS and pathologically associated proteins found at MERCS are important players in various neurodegenerative disorders (Paillusson et al., 2016). In amyotrophic lateral sclerosis (ALS), a neuromuscular neurodegenerative disorder that causes gradual loss of motor neurons, overexpression of TDP-43, one of the thought initiators of disease, dampens the ER to mitochondria apposition *via* the VAPB-PTPIP51 tethering complex (Stoica et al., 2014). Dopaminergic neuronal loss and cytosolic α-synuclein aggregates have been detected in Parkinson’s disease (PD) patients’ brains. α-synuclein has been localized at MERCS, and mutant forms of this protein disrupt the ER to mitochondria apposition (Paillusson et al., 2017). Huntington’s disease (HD) is an autosomal dominant neurodegenerative condition in which mutant Huntingtin protein (Htt) accumulates in the nucleus. Htt disrupts ER–mitochondrial interactions, resulting in the dampening of Ca^2+^ signaling, in the striatum of HD mutant mice (Cherubini et al., 2020).

### 3.1 Mitochondria–Endoplasmic Reticulum Contact Sites in Alzheimer’s Disease Pathology

The scientific community has given special attention to MERCS in the context of AD pathogenesis, with evidence pointing to the increased ER to mitochondria apposition as a key factor in AD pathogenesis (Area-Gomez et al., 2018). Area-Gomez et al. (2009) pioneered the discovery of the presence of APP, as well as the γ-secretase components PS1 and PS2, in the MERCS-enriched fraction of mouse brains. Furthermore, studies on SH-SY5Y cells carrying PS2 FAD mutations (T122R) showed for the first time increased ER to mitochondria apposition and Ca^2+^ shuttling (Zampese et al., 2011). Indeed, PS2 was shown to interact with the negative regulator of MERCS, Mfn2 (Filadi et al., 2016). An increase in the MERCS number was observed in fAD fibroblasts and sAD fibroblasts (Area-Gomez et al., 2012), mouse and rat primary neurons treated with Aβ (Hedskog et al., 2013; Calvo-Rodríguez et al., 2016; Calvo-Rodriguez et al., 2019), SH-SY5Y cells overexpressing APP (Del Prete et al., 2017), and the hippocampus of APP knock-in *App* ^*NL-F*^ and *App* ^*NL-G-F*^ mice (Leal et al., 2020). We have also recently demonstrated that inhibiting oAβ selectively, using the Aβ-neutralizing scFvA13 antibody, reversed the increase in MERCS number, implying that oAβ peptides have a direct and specific effect on contact dynamics (Leal et al., 2020). Further evidence has been emerging connecting MERCS and AD. For example, membrane cellular fractionation experiments on adult mouse brains reveal that Aβ is present in the MERCS-enriched fraction along with the APP-processing machinery including β-secretase and active γ-secretase (Schreiner et al., 2015; Del Prete et al., 2017). The unprocessed APP fragment C99 has been localized at the MAM and shown to increase the MERCS activity and apposition (Pera et al., 2017). Not only are these components present at MERCS but also MERCS themselves are capable of modulating Aβ production. MFN KO cells and conditional knockdown of contact regulator Mfn2 was accompanied by decreased γ-secretase activity (Area-Gomez et al., 2012) and Aβ production (Leal et al., 2016). The exact mechanism linking MERCS and Aβ production is currently unknown.

To date, limited studies have analyzed the role of tau in ER–mitochondria contacts. Tau has been localized at both the mitochondria and ER (Perreault et al., 2009; Cieri et al., 2018a). Mice overexpressing P301L tau showed upregulated juxtaposition between the two organelles in spinal cord motor neurons (Perreault et al., 2009). One study showed that the truncated caspase 3-cleaved 2N4RΔC20 tau protein induced fibrillation and seeding of wild-type (WT) tau, leading to the increased ER to mitochondria proximity (Cieri et al., 2018b). Whether tau affects MERCS apposition itself remains elusive; target validation studies should address this important question.

So far only two studies have reported the decreased ER to mitochondria proximity in AD models. Lower MERCS apposition has been shown in transgenic rats overexpressing APP at a 10-nm distance using a FRET system of proximity detection, while MERCS length investigated using the EM was also reported; however, number of contacts or distance of contacts were not investigated using the EM (Martino Adami et al., 2019). Accordingly, in the postmortem sAD brain cortex using a proximity ligation assay (PLA), fewer VAPB-PTPIP51 appositions were detected in early to mid-Braak stages (Lau et al., 2020a). These conflicting results might be due to less sensitive techniques, which measure indirect ER to mitochondria apposition, compared with transmission electron microscopy (TEM) studies.

Regardless of these discrepancies, it appears that MERCS play a substantial role in the progression of AD pathology, with most studies reporting increased ER to mitochondria contacts as a pathological phenotype in AD models.

### 3.2 Increased Contacts in Alzheimer’s Disease—Beneficial or Harmful?

MERCS role in AD pathogenesis is a relatively recent field, and although extensive research has been carried out in the last decade to understand the basic mechanism and nature of MERCS, several questions remain withstanding. Of relevance, the increased proximity between the ER and mitochondria in AD has been shown to have both beneficial and deleterious effects in different models. Indeed, it is important to note that MERCS are not static entities; they are dynamic structures that change depending on the bioenergetic needs of the cell (Sood et al., 2014). We believe that increased MERCS in AD might be an early compensatory mechanism to offset changes in the cellular environment and increase ATP production to sustain its compromised function. This hypothesis agrees with a recent study showing that increasing the ER to mitochondria apposition restores climbing ability and increased lifespan in Αβ_42_ overexpressing flies (Garrido-Maraver et al., 2020). Furthermore, we have, in recent studies, found that increased mitochondrial activity precedes amyloid deposition in *App* ^*NL-G-F*^ mice (Naia et al., unpublished) and in *App* ^*NL-F*^ embryonically derived primary neurons (Dentoni et al., unpublished), suggesting that increased MERCS and hypermetabolism are an early feature of the disease. However, on the other hand, a sustained increase in MERCS can have deleterious effects on the cell, eventually leading to apoptosis (Csordas et al., 2006). Increased retention of Ca^2+^ was detected in AD models implying that high and sustained levels of Ca^2+^ can induce mitochondrial matrix overload and Ca^2+^-activated mitochondrial permeability transition pore opening, resulting in age-associated cognitive decline in AD models (Jadiya et al., 2019). Furthermore, a recent preprint has shown that artificially increasing MERCS block mitochondrial movement in axons, resulting in deformed neuromuscular junctions in flies (Hewitt et al., 2020). From this evidence, we believe that when an early upregulation of MERCS might be necessary to boost bioenergetics, as pathology progresses, this compensatory mechanism might lead to apoptosis due to overactive Ca^2+^ shuttling. Therefore, it is fundamental to assess how modulation of contacts at specific timepoints of pathogenies could be beneficial for the disease. Due to the conflicting evidence in the literature reported, it would be important to test strategies that both increase and decrease contacts and observe how this relates to changes in the pathogenesis of the disorder, its progression, and neuroprotective properties of specific MERCS modulators. Indeed, it might be necessary to use strategies to increase MERCS early while pharmacological blockage of the ER to mitochondria apposition might be necessary for patients with advanced AD.

## 4 Use of Small Molecules to Modulate Mitochondria–Endoplasmic Reticulum Contact Sites’ Function and Restore Neuronal Homeostasis in Alzheimer’s Disease

**FIGURE 2:**
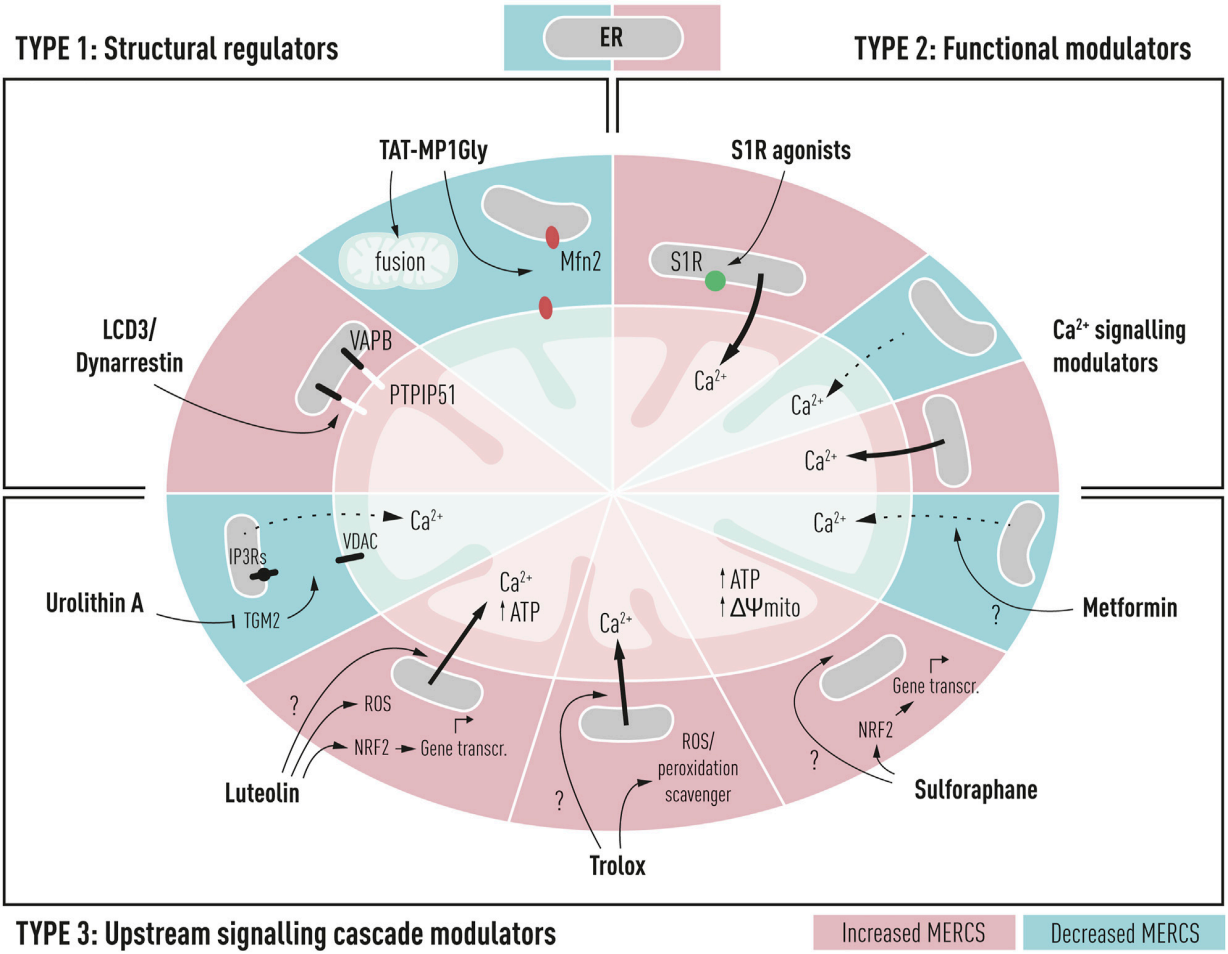
Schematic representation of potential drug targets for the modulation of mitochondria–ER contacts in Alzheimer’s disease and their cellular and mitochondrial effects. TYPE 1: structural regulators such as LCD3/Dynarrestin and TAT-MP1Gly can interact directly with structural regulators of contacts such as PTPIP51 and Mfn2 resulting in increased or decreased proximity between the two membranes. TYPE 2: functional modulators could be used to alter the ER to mitochondria functional proximity, through modulation of Ca^2+^ shuttling between the ER and mitochondria; such compounds include sigma-1 receptor agonists (S1R) and Ca^2+^ signaling modulators targeting IP3Rs, VDAC, or MCU complex. **TYPE** 3: upstream signaling cascade modulators alter proximity between two membranes, but their specific target is unknown. These compounds could affect upstream signaling cascades, leading to the alteration of MERCS proximity. These compounds include metformin, sulforaphane, Trolox, luteolin, and urolithin A.

Developing molecules to correct and/or modulate the mitochondria–ER interplay can unleash unique therapeutic opportunities in AD. Recently, MERCS modulators have been categorized based on either their interaction with MERC-resident proteins, protein expression levels, or upstream signaling cascades (Magalhães Rebelo et al., 2020). For this review, we have categorized MERCS modulators slightly different based on 1) their interaction with tethering or contact regulators, 2) interaction with functional modulators at MERCS, and 3) upstream signaling cascades. We believe that there is an opportunity of targeting MERCS with different small molecules to modulate its morphology and/or functions. In doing so, we could stimulate downstream cascades that can either promote cellular homeostasis or sensitize to current AD therapies or help restore cellular homeostasis in the prodromal stage of AD. In the following section, we surveyed small molecules with MERCS modulatory potential both structurally and functionally (MERCS modulators), aiming to restore neuronal homeostasis in AD, summarized in Figure 2. In addition, we collected the molecular structure, associated MERCS molecular target, and its observed effect on MERCS from different publications (Table 1). Although some molecules have not yet been tested in AD models, we believe their proven MERCS role or their interaction with MERCS components has the potential to restore neuronal homeostasis. We set a stringent criterion to select molecules with proven MERCS modulatory effect or molecules having an extremely likely effect on this subcellular region, due to their interaction with key tethering proteins at MERCS. Indeed, analyzing the literature on the subject, several molecules have been shown to alter levels of tethering and non-tethering proteins at MERCS; however, whether changes in these proteins translate to changes in structures or functions remain to be debated.

**TABLE 1:** Examples of modulators targeting MERCS.

Compound	Structure/sequence	Associated MERCS molecular target	Associated MERCS effect	Reference
LCD3/Dynarrestin	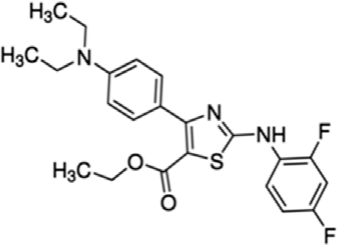	Unknown. Its effects are dependent on PTPIP51	• Potential target, affecting MERCS tethering	Dietel et al. (2019)
TAT-MP1Gly	DIAEAVRLIMDSLHMAAR	Directly targets Mfn2	• Restores Mfn2 active configuration	Franco et al. (2016a)
			• Potential to target tethering	
Pridopidine	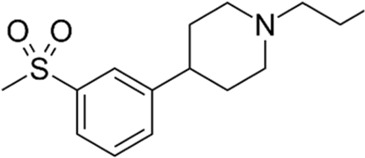	Sigma σ1 receptor agonist	• Restores MERCS connectivity and Ca^2+^ signaling	Naia et al. (2021a)
			• Restores mitochondrial respiration, dynamics, and motility	
Metformin	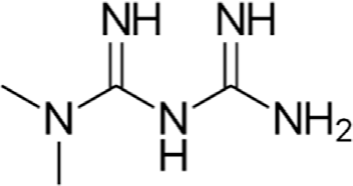	Unknown	• Decreases ER–mitochondria apposition	Angebault et al. (2020)
			• Decreases mitochondrial Ca^2+^ content and improvement in complex I respiration	
Sulforaphane	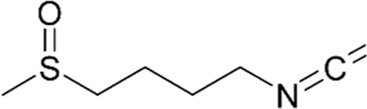	Unknown	• Indirectly restores MERCS interactions	Tubbs et al. (2014); Tian et al. (2021)
			• Decreases ER stress and increases ER to mitochondria Ca^2+^ shuttling, ΔΨm, and ATP synthesis	
Trolox	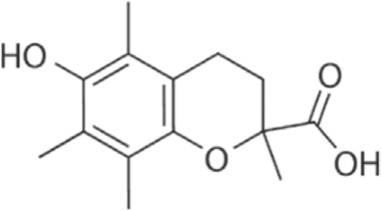	Unknown	• Restores MERCS function and Ca^2+^ levels	Rodríguez et al. (2020)
Luteolin	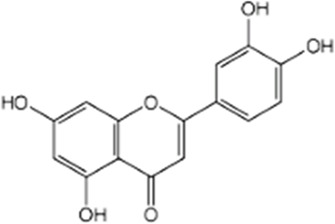	Unknown	• Increases MERCS apposition, Increases in ATP production and mitochondrial bioenergetics	Naia et al. (2021b)
Urolithin A	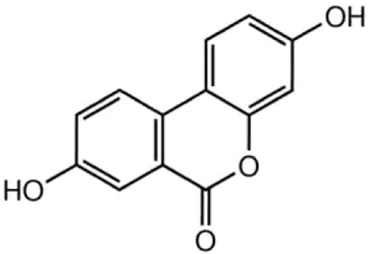	Urolithin A reduces TGM2 levels and MERCS formation, by affecting the AIP–AhR transcription complex	• Decreases MERCS apposition, ER to mitochondria Ca^2+^ shuttling, and mtROS production	Lee et al. (2021)

### 4.1 Structural Regulators

#### 4.1.1 LDC3/Dynarrestin

LDC3/Dynarrestin is a small aminothiazole inhibitor of cytoplasmic dynein 1 and 2 (Höing et al., 2018). The molecule interferes with the Hedgehog pathway by the inhibition of dynein, affecting the endosome movement and mitosis. PTPIP51 can activate the MAPK pathway *via* Raf1 coupling. One study has shown that the knockdown of PTPIP51 abolishes the MAPK stimulating effect of LDC3/Dynarrestin normally induced by the PTPIP51/14-3-3/Raf1 interactome (Brobeil et al., 2017).

##### 4.1.1.1 Mitochondria–Endoplasmic Reticulum Contact Sites’ Modulation

In a recent small high-throughput screen, LCD3/Dynarrestin was shown to possess a high affinity to PTPIP51 (Dietel et al., 2019). PTPIP51 acts as a linker to bind to VAPB and regulate Ca^2+^ homeostasis and autophagy, Figure 1 (Stoica et al., 2014; Gomez-Suaga et al., 2017). In the original study, LCD3/Dynarrestin increased the PTPIP51 interaction with VAPB, having its highest effect at high concentrations of 5 and 50 µM for 24–48 h, hinting to a role of this molecule in modulating MERCS. Whether this molecule affects the MERCS ultrastructure and functions is not known; however, an MTT assay was performed to assess the mitochondrial metabolic activity upon treatment. No changes were seen in the MTT assay up to 5 µM concentration, while at 50 µM of LCD3/Dynarrestin for 24 h impaired the mitochondrial metabolic activity (Dietel et al., 2019).

##### 4.1.1.2 Therapeutic Potential in Alzheimer’s Disease and Clinical Trial

Modulation of PTPIP51 with LCD3/Dynarrestin could modify related signaling pathways in AD and create a “cascade-like” Ca^2+^ homeostasis, due to its potential in modulating the ER to mitochondria apposition. The increased interaction between PTPIP51 and VAPB is thought to result in a more stable ER–mitochondria juxtaposition. Although increased interaction between the ER and mitochondria has been observed in AD, in early AD postmortem brains, lower levels of VAPB and decreased proximity ligation assay immunoreactivity between VAPB and PTPIP51 have also been reported (Lau et al., 2020b). As a result, this molecule may have a beneficial effect in stabilizing such couple interactions or further supporting metabolism through ER to mitochondria Ca^2+^ shuttling. However, in the original study, the authors also argue that LCD3/Dynarrestin regulates this interaction for longer incubation times and at high concentrations, suggesting that the increased PTPIP51 and VAPB interaction may be caused by secondary mechanisms (Dietel et al., 2019). Future studies on this molecule shall shed light on these unanswered questions.

#### 4.1.2 TAT-MP1Gly

TAT-MP1Gly is a cell-permeant peptide, based on the residues 367–384 of Mfn2 and the residues 47–57 of the HIV transactivator protein, capable of modifying Mfn2 structural conformation and thought to promote Mfn2-dependent mitochondrial fusion (Franco et al., 2016a).

##### 4.1.2.1 Mitochondria–Endoplasmic Reticulum Contact Sites’ Modulation

Charcot–Marie–Tooth disease type 2A (CMT2A) is a hereditary neurological disorder characterized by motor and sensory neuropathy of the peripheral nervous system, leading to foot and lower leg muscle weakness and paralysis. Mfn2 autosomal dominant mutations cause pathological mitochondrial fragmentation in CMT2A. TAT-MP1Gly treatment for 24 h reversed mitochondrial fragmentation, clumping, and mitochondrial depolarization *in vitro* from mouse neurons expressing either the artificial GTPase-deficient Mfn2K109A mutant or the naturally occurring human Charcot–Marie–Tooth disease type 2A (CMT2A) GTPase mutant, Mfn2T105M (Franco et al., 2016b). Several lines of evidence point to impaired ER–mitochondria tethering both in mouse and human fibroblasts as a pathological feature of Mfn2-mediated CMT2A, resulting in decreased ER to mitochondria apposition and Ca^2+^ shuttling (Bernard-Marissal et al., 2019).

##### 4.2.2.2 Therapeutic Potential in Alzheimer’s Disease and Clinical Trial

In AD, Mfn2 levels have been shown to be downregulated both *in vitro*, *in vivo*, and in postmortem brains (Wang et al., 2009; Leal et al., 2020). Functionally, AD mitochondria appear smaller and fragmented likely due to Mfn2 loss of function (Wang et al., 2009). Mfn2 has also been implicated in MERCS dynamics *in vitro* in AD (Leal et al., 2020). Similar to CMT2A, AD is characterized by the widespread fragmented mitochondria, and enhancing the Mfn2 function might have a positive effect on mitochondrial dynamics (Wang et al., 2020). As Mfn2 plays an important role either as a tethering or negative regulator of contacts, conformational changes in Mfn2 could likely correct changes in MERCS and restore mitochondrial dynamics in AD models. No studies so far have assessed the MERCS-dependent role of these peptides in AD or in CM2A. We believe that Mfn2 modulation could have benefits for AD by both reestablishing mitochondrial dynamics and either directly or indirectly restoring MERCS homeostasis. As we believe Mfn2 plays as a negative regulator at MERCS, these peptides might enhance the Mfn2 spacer activity at MERCS, normalizing its physiology.

### 4.2 Functional Modulators

#### 4.2.1 Sigma-1 Receptor Agonists

The sigma-1 receptor (S1R) is enriched at MERCS, where it stabilizes IP3R, regulating the IP3R-mediated ER to mitochondrial Ca^2+^ transfer (Figure 1) (Hayashi and Su, 2007). Several S1R agonists have been tested and shown to have positive effects on neurodegenerative disorders including pridopidine (Ryskamp D. A. et al., 2019).

##### 4.2.1.1 Mitochondria–Endoplasmic Reticulum Contact Sites’ Modulation

In HD models where ER to mitochondria apposition is decreased, pridopidine promotes normal IP3R-dependent Ca^2+^ release, as well as activation of critical Ca^2+^-regulating genes (Eddings et al., 2019). Furthermore, pridopidine treatment restored MERCS connectivity and improved co-localization of IP3R3 and its chaperone S1R within mitochondria in striatal neurons isolated from YAC128 mice. By affecting the MERCS signaling platform, mitochondrial respiration, dynamics, and motility were recovered in pridopidine-treated YAC128 neurons (Naia et al., 2021a). In AD models such as PS1-KI mice, administration of pridopidine stabilized mushroom spines *in vivo* and *in vitro* through a Ca^2+^-ER-dependent mechanism (Ryskamp et al., 2017), suggesting the mechanistic involvement of MERCS; however, no study in AD has made the connection between MERCS dynamics and pridopidine treatment. Although it is known that pridopidine affects MERCS-resident S1R, whether this agonism influences direct or indirect MERCS through indirect modulation of Ca^2+^ signaling remains unexplored.

##### 4.2.1.2 Therapeutic Potential in Alzheimer’s Disease and Clinical Trials

Interestingly, S1R polymorphisms have been found to affect the likelihood of acquiring AD, which working synergistically with apolipoprotein E (APOE) increases the risk of AD (Huang et al., 2011). An early postmortem investigation found that the S1R was reduced in the CA1 region of the hippocampus in AD patients. Positron emission tomography (PET) using a radioligand for the receptor was used confirming its depletion in the early stages of AD (Mishina et al., 2008). Among other functions, S1R has been reported to take part in neuromodulation and neuroplasticity, stressing its potential role in affecting memory and cognition (reviewed in (Maurice et al., 2006; Kourrich et al., 2012). Concordantly, different S1R modulators such as pridopidine, N-n-propyl-3-(3-hydroxyphenyl) piperidine (3-PPP), and AF710B (ANAVEX 3–71) displayed neuroprotective properties in animal models of AD, reviewed in Ryskamp D. et al. (2019). Several ongoing trials for HD such as PROOF-HD (Phase 3) (Reilmann et al., 2021) and HEALEY ALS (Phase 2–3) (Paganoni et al., 2022) have been spurred by promising results of pridopidine *in vitro* and *in vivo*. AD pridopidine itself is not currently being tested; nonetheless, other S1R agonists including blarcamesine (phase II/III) (Hampel et al., 2020), edonerpic (phase II) (Schneider et al., 2019), dextromethorphan formulations AVP-786 (phase III) (Cummings et al., 2015), AVP-923 (phase IV) (Fralick et al., 2019), and AXS-05 (phase II-III) (Wilkinson and Sanacora, 2019) are undergoing clinical trial validation. In summary, these studies suggest that pridopidine and other S1R agonists could act as disease-modifying and neuroprotective agents in AD by stimulating MERCS-associated functions. In the context of AD, we believe such molecules could be used in the early stages to sustain MERCS-mediated bioenergetics and ATP production.

#### 4.2.2 Ca^2+^ Signaling Modulators

The functional bridge shuttling Ca^2+^ between the ER and mitochondria consisting of IP3R-Grp75-VDAC and the mitochondrial Ca^2+^ uniporter (MCU) is essential for the regulation of Ca^2+^ dynamics and associated MERCS function including bioenergetics (Rizzuto and Pozzan, 2006), autophagy (Gomez-Suaga et al., 2017), mitochondrial dynamics (Friedman et al., 2011b), and vesicle release (Dentoni et al., 2022).

##### 4.2.2.1 Mitochondria–Endoplasmic Reticulum Contact Sites’ Modulation and Therapeutic Potential in Alzheimer’s Disease and Clinical Trials

Several studies have shown potential neuroprotective effects of blockage of MERCS-mediated Ca^2+^ shuttling. Indeed, blocking IP3Rs and MCU has been the most used strategies to influence Ca^2+^ in this nanodomain (Gomez-Suaga et al., 2017; Naia et al., 2021a). This has been thoroughly reviewed in several publications (Magalhães Rebelo et al., 2020; Leal and Martins, 2021; Rodríguez et al., 2022). Briefly, various strategies can be implemented targeting the Ca^2+^ shuttling bridge at MERCS:

•IP3Rs: fAD PS1 mutations have been shown to potentiate the IP3R-mediated Ca^2+^ release (Leissring et al., 2001). In fAD PS1 mice, knockdown of IP3R1 normalized disrupted Ca^2+^ signaling, alleviating mutant PS-linked fAD pathogenesis (Shilling et al., 2014). IP3R hyperactivity in AD suggests that inhibiting the IP3R function might have neuroprotective effects. IP3R inhibitors such as xestospongin B and C are shown to modulate MERCS-mediated functions such as autophagy (Gomez-Suaga et al., 2017), vesicle release (Dentoni et al., 2022), bioenergetics, and Ca^2+^ homeostasis (Cardenas et al., 2010b).
•VDAC: VDAC is a critical protein in mitochondria-mediated apoptosis and acts as a mitochondrial gatekeeper, controlling metabolic dynamics between the mitochondria and the cytoplasm (Shoshan-Barmatz et al., 2018). Its interaction with AD hallmarks such as Aβ (Smilansky et al., 2015), phopsho-tau (Manczak and Reddy, 2012), and γ-secretase (Hur et al., 2012) modifies its properties. Indeed, the Aβ interaction with VDAC resulted in a two-fold increase in channel conductance, resulting in mitochondrial dysfunction and apoptosis (Smilansky et al., 2015). Several classes of molecules have been shown to influence VDAC opening directly or through its interactions such as AKOS-022, hesperidin, and VBIT-3/4 reviewed in Magrì et al. (2018).
•MCU: MCU has been widely recognized as a potential target for AD. Aβ load has been shown to increase cytosolic Ca^2+^, resulting in mitochondrial Ca^2+^ overload in APP/PS1 mouse models. MCU has a pivotal role in this pathway, as inhibiting MCU with Ru360 blocks mitochondrial Ca^2+^ overload (Calvo-Rodriguez et al., 2020). Excessive Ca^2+^ uptake by mitochondria can cause mPTP opening, caspase activation, and neuronal cell death (Hurst et al., 2017). Drugs such as the recently identified cell-permeable MCUi4 and MCUi11 could be used to avoid increased mitochondrial Ca^2+^ matrix accumulation (Di Marco et al., 2020). Decreased activation of MCU and its components, however, have been shown to enhance neuronal damage in mouse neurons and fibroblasts (Verma et al., 2017). A recent review has thoroughly assessed how MERC-dependent MCU activation could be neuroprotective in models of neurodegeneration, highlighting molecules such as kaempferol as potential agents to prevent neurodegeneration (Rodríguez et al., 2022).

Developing compounds that target this MERCS Ca^2+^ axis may be challenging due to the multifunctional role of Ca^2+^ in the cells and the plethora of mechanisms it is involved in. What is challenging about these strategies is to find a balance between enough ER to mitochondria Ca^2+^ shuttling to sustain bioenergetics and not too much interaction that would have deleterious effects such as ROS production and induction of apoptosis.

### 4.3 Upstream Signaling Cascade Modulators

#### 4.3.1 Metformin

Metformin is an antihyperglycemic drug reducing the glucose output and increasing the insulin-mediated glucose uptake. The molecular targets of metformin include the mitochondrial complex I and other enzymes modulated by the altered energy metabolism, including AMPK signaling, reviewed in Pernicova and Korbonits (2014).

##### 4.3.1.1 Mitochondria–Endoplasmic Reticulum Contact Sites’ Modulation

Studies on the role of metformin in modulating the ER to mitochondria juxtaposition in neurons are scarce. In cardiomyocytes, metformin was shown to have a cardioprotective effect at MERCS. In a recent study, the number of MERCS calculated as the proximity between IP3R1 and VDAC1 was upregulated in Duchenne muscular dystrophy (DMD) cardiomyocytes. IP3R1, as well as MCU and its regulatory subunit, MICU1, expression was upregulated in the DMD heart. In this mouse model, increased MERCS was also associated with an increase in the mitochondrial Ca^2+^ uptake and complex I dysfunction and ROS production (Angebault et al., 2020), also reported in AD. Treatment of DMD mice for 1 month with metformin improved complex I-driven respiration, normalizing MERCS number, decreasing MICU1 expression, and mitochondrial Ca^2+^ concentration (Angebault et al., 2020). Whether these effects on MERCS are direct or indirect remains unknown; target validation analysis could shed light on metformin-dependent pathways and mechanisms.

##### 4.3.1.2 Therapeutic Potential in Alzheimer’s Disease and Clinical Trials

Insulin resistance and diabetes are becoming well recognized as risk factors in the development of dementia and AD (Burillo et al., 2021). Metformin’s potential by acting on mitochondrial metabolism and insulin signaling is the basis for its use for neurodegenerative disorders and AD. Metformin use is linked to a decreased risk of cognitive impairment in T2DM patients (Teng et al., 2001). Studies show that diabetics taking metformin had a decreased likelihood of developing AD than diabetics taking other glucose-lowering drugs (Chin-Hsiao, 2019). Metformin is currently being used as a neuroprotective agent in MCI patients in clinical trials in phases II and III (NCT0498666). By decreasing the ER to mitochondria apposition, these drugs could be used in later stages of the disease to prevent excessive Ca^2+^ shuttling, hence counteracting negative effects of overtly active MERCS such as ROS production and apoptosis initiation. Combined with MERCS off-target effects, metformin has the potential and is currently being considered as a powerful neuroprotector in AD.

#### 4.3.2 Sulforaphane

Sulforaphane (SFN) is a small lipophilic isothiocyanate abundantly found in green vegetables such as broccoli, with higher bioavailability in the bloodstream than other phytochemicals (Hanlon et al., 2008). It has been showed that SFN can reduce glucose production and improve glucose control in patients with type 2 diabetes (Axelsson et al., 2017). Previous studies have showed that SFN induces nuclear translocation of the nuclear factor erythroid 2-related factor 2 (Nrf2) and binding to G6pc and PEPCK to suppress gluconeogenesis (Heiss et al., 2013). Since activation of Nrf2 protects against oxidative stress and supports the structural and functional integrity of mitochondria (Dinkova-Kostova and Abramov, 2015).

##### 4.3.2.1 Mitochondria–Endoplasmic Reticulum Contact Sites’ Modulation

No studies so far have analyzed the role of this molecule in MERCS modulation in neuronal models. However, SFN has been shown to have MERCS-positive effects in hepatocytes. Indeed, MERCS have been shown to be intercellular hubs for hepatic metabolism. In insulin resistance models, studies have shown MERCS to be either downregulated or upregulated (Arruda et al., 2014; Tubbs et al., 2014). Regarding MERCS modulation, SFN improved disrupted ER–mitochondria interactions in primary mouse hepatocytes isolated from mice with genetically induced obesity, resulting in increased IP3R1–VDAC proximity, increased MERCS protein levels, and decreased ER stress (Tubbs et al., 2014). In nonalcoholic fatty liver disease (NHFLD) mouse models where the ER to mitochondria apposition is shown to be upregulated, SFN maintained Ca^2+^ homeostasis at MERCS by regulating the Ca^2+^ flux *via* Ca^2+^ channels IP3R and MCU. When MERCS numbers were not actively assessed post-treatment in this study, ΔΨm and ATP synthesis were upregulated post-SFN treatment (Tian et al., 2021).

##### 4.3.2.2 Therapeutic Potential in Alzheimer’s Disease and Clinical Trials

Recently, the potential of SFN as a neuroprotective agent has been summarized showing its potential as a small-molecule modulator in different animal models of AD (Kim, 2021). The SFN neuroprotective potential has been considered for the treatment of prodromal and mild AD, which is currently recruiting (NCT04213391). Other analogs of SFN, such as Avmacol under clinical investigation, have been shown to modulate the symptoms of autism (Bent et al., 2018). Nevertheless, the mechanism in which SFN improves ER–mitochondria interactions is an open field that requires further investigation. We speculate on the use of SFN or its analogs could activate the Nrf2 pathway, improve ER–mitochondria interactions, activate the transcriptional regulation of cytoprotective genes, and regulate concomitant pathways such as inflammation, mitochondrial dynamics, and cellular metabolism.

#### 4.3.3 Trolox

Trolox (6-hydroxy2,5,7,8-tetramethylchroman-2-carboxylic acid) is a small water-soluble vitamin E-derived antioxidant. As a lipid peroxidation scavenger, Trolox aids in the stabilization of cellular membranes (Lúcio et al., 2009).

##### 4.3.3.1 Mitochondria–Endoplasmic Reticulum Contact Sites’ Modulation

Several lines of evidence point to Trolox as a potential MERCS modulator. Earlier studies have shown that Trolox treatment increases the protein levels of Mfn2 in Chinese hamster ovary (CHO) cells (Distelmaier et al., 2012). Another study showed chronic Trolox treatment to increase mitochondrial complex I activity, reduce ROS levels, and restore cytosolic Ca^2+^ ATP handling and ΔΨm in primary skin fibroblasts isolated from children with NADH deficiency (Koopman et al., 2008). A study showing the direct role of this drug on MERCS reported that in the neuroblastoma cell model of Friedreich ataxia (FRDA), Trolox could restore MERCS and increase Ca^2+^ shuttling between the ER and mitochondria (Rodríguez et al., 2020). Friedreich ataxia (FRDA) is a neurodegenerative disorder leading to dorsal root ganglia degeneration, caused by mutations of the *FXN* gene, which encodes frataxin (FXN), a mitochondrial protein found in the IMM. Modulation of MERCS through Marf knockdown (Mfn2 *D. melanogaster* homolog) has been shown to be beneficial in frataxin-deficient glial cells, likely through increased ER to mitochondria apposition which was not evaluated in this study (Edenharter et al., 2018). Similarly, Trolox restoration of Ca^2+^ levels through the ER to mitochondria increased juxtaposition was sufficient to reverse the defects caused by frataxin deficiency in *D. melanogaster* (Rodríguez et al., 2020).

##### 4.3.3.2 Therapeutic Potential in Alzheimer’s Disease and Clinical Trial

The efficacy of vitamin E and its derivatives, such as Trolox, as a potential treatment intervention for AD has been tested in several randomized studies (Regner-Nelke et al., 2021). Trolox can also protect against neurotoxicity caused by Aβ and inhibit glycogen synthase kinase 3 (GSK 3), a protein in neurofibrillary tangle development (Muñoz et al., 2002; Radesäter et al., 2003). Despite this, evidence for vitamin E’s therapeutic effectiveness in the treatment of AD remains unclear. Despite strong evidence of increased ROS in AD etiology and lower circulating vitamin E levels, clinical trials examining vitamin E as a treatment have yet to show conclusive results (Regner-Nelke et al., 2021). When no clinical studies have been conducted with Trolox specifically for AD, studies have shown that a combination of donepezil and Trolox has been tested as multifunctional agents for AD. These compounds had moderate to good inhibitory actions against acetylcholinesterase (AChE) and monoamine oxidase B (MAO-B), as well as significant antioxidant properties (Cai et al., 2017). These compounds have shown promising improvements in cognition and spatial memory *in vivo*. The additional effects of Trolox on MERCS could stabilize and promote bioenergetics mechanism, decreasing ROS production by effectively coupling respiration and resulting in an overall improvement in the AD phenotype.

#### 4.3.4 Luteolin

Luteolin is a natural flavone that has been found to have potent antioxidant and anti-inflammatory activities (Nabavi et al., 2015). One of the most common protective mechanisms of luteolin is to increase the binding of Nrf2 to the antioxidant responsive element (ARE) (Wruck et al., 2007; Lin et al., 2010). The Nrf2–ARE pathway is a mechanism of defense which reduces oxidative stress and neuroinflammation promoting neuronal homeostasis. In AD, it has been shown that the Nrf2–ARE pathway is disrupted mainly due to decreased Nrf2 expression (Ramsey et al., 2007).

##### 4.3.4.1 Mitochondria–Endoplasmic Reticulum Contact Sites’ Modulation

In addition to luteolin’s well-known properties, we recently reported a new mechanism for luteolin-induced neuroprotection through MERCS. Our laboratory established a neuronal cell-based high-throughput screen to search for mitotherapeutics and identified luteolin as one hit. Low micromolar concentrations of luteolin increased bioenergetics in primary mouse neurons and isolated mouse brain mitochondria and crude synaptosomal preparations containing MERCS (Naia et al., 2021b). We identified ER to mitochondrial Ca^2+^ shuttling as a mechanism promoted by luteolin, increasing the Krebs cycle activity and consequently mitochondrial respiration and ATP production. Luteolin enhanced MERCS through an IP3Rs-dependent mechanism as inhibition of IP3Rs through xestospongin C treatment hindered luteolin-induced ATP production. Luteolin administration also partially recovered mitochondrial and locomotory activities in *C. elegans* and primary neuron models expressing the mutant huntingtin (Naia et al., 2021b).

##### *4.3.4.2 Therapeutic Potential in Alzheimer’s Diseas*e *and Clinical Trial*

Several *in vitro* and *in vivo* studies have reported positive effects of luteolin on reducing ROS production (Dragicevic et al., 2011), restoring ATP levels (Dragicevic et al., 2011), increasing synaptic marker expression (Xu et al., 2013), reducing pathological hallmarks of AD and decreasing Aβ production and tau phosphorylation (Sawmiller et al., 2014), and restoring cognitive functions (Wang et al., 2016). Interestingly, luteolin was also reported to increase the sacro/endoplasmic reticulum Ca^2+^ ATPase 2a (SERCA2a) activity, which in turn influences Aβ generation (Green et al., 2008; Zheng et al., 2015), pointing to its Ca^2+^-modifying properties in AD. Luteolin has been used in clinical phase 2 trials as a dietary supplement for 4 months with positive outcomes increasing behavioral and social interactions in subjects with autism spectrum disorder (ASD) (Taliou et al., 2013). A recent clinical trial assessing the effect of luteolin on memory in healthy subjects was started in January 2020 but halted due to the COVID-19 pandemic (https://clinicaltrials.gov/, NCT04468854); hence, this compound is currently being considered for the treatment of neurodegenerative disorders in humans.

#### 4.3.5 Urolithin A

Urolithin A is a gut microbial product, metabolized from ingested ellagitannins and ellagic acid (D’Amico et al., 2021). Surprisingly, the conversion of dietary precursors to urolithin A does not occur equally in all individuals, depending on specific gut microbiota composition (Cortés-Martín et al., 2020). Due to its anti-inflammatory and neuroprotective characteristics in preclinical AD models, PD models, diabetes mellitus, ischemic neuronal injury, and normal aging, urolithin A has received considerable attention recently (D'Amico et al., 2021).

##### 4.3.5.1 Mitochondria-Endoplasmic Reticulum Contact Sites’ Modulation

The role of urolithin A in modulating MERCS was unknown until very recently. In a hyperglycemia cell model, characterized by increased ER to mitochondria Ca^2+^ shuttling and increased mtROS production and Aβ production, transglutaminase 2 (TGM2) increased MERCS by stabilizing the IP3R–Grp75–VDAC interaction (previously discussed in Section 2.2 ER to mitochondria Ca^2+^ shuttling). Indeed, TGM2 knockdown reverted pathology in cells under hyperglycemic stress (Lee et al., 2021). Similar to TGM2 knockdown, urolithin A reduced TGM2-dependent MERCS formation and ROS and Aβ production by affecting the AIP–AhR transcription complex, resulting in reduced TGM2 levels (Lee et al., 2021).

##### 4.3.5.2 Therapeutic Potential in Alzheimer’s Disease and Clinical Trial

From the aforementioned data, Urolithin A has emerged as a promising candidate to disengage stable MERCS in conditions in which the ER to mitochondria apposition is upregulated such as in AD and diabetes, a major risk factor for developing dementia. In AD animal models such as APP/PS1 mice, urolithin A treatment improved cognition and enhanced neurogenesis (Gong et al., 2019). Furthermore, urolithin A attenuated Aβ deposition, microgliosis, and astrocytosis in the cortex and hippocampus (Fang et al., 2019; Gong et al., 2019). Urolithin A is capable of persisting in the plasma and crossing BBB, making it an ideal neuroactive compound (Gasperotti et al., 2015; Kujawska et al., 2020). Clinical trials on this compound are limited; however, some are currently ongoing to assess the bioavailability of urolithin A in patients’ blood (NCT04985630), its protective role in prostate cancer (NCT03535675), and to boost muscle endurance (NCT04783207).

## 5 Current Challenges and Future Perspectives to Develop Mitochondria–Endoplasmic Reticulum Contact Sites’ Function and Modulators

Experimental efforts spanning over the past decade have revealed MERCS as a new player in AD pathogenesis. We believe that the therapeutic potential of MERCS modulators requires further translational research to overcome several challenges.

One of the most ambitious challenges regarding pharmaceutical modulation of MERCS is the difficulty of finding the precise conditions in which small-molecule modulators “fine-tune” MERCS to achieve neuronal balance. For example, the “fine-tuning” of MERCS to increase the Ca^2+^ flux and promote Krebs cycle activity would promote neuronal homeostasis. However, excessive Ca^2+^ flux can activate ROS production and apoptosis pathways, leading to cell death (Csordas et al., 2006). Therefore, increasing many or decreasing very few MERCS-associated functions can create an adverse effect and be deleterious for neuronal homeostasis. Finding MERCS modulators that could sense the bioenergetics needs of the cell, such as MERCS do under physiological conditions, would be fundamental to reestablish neuronal homeostasis. As previously mentioned, understanding whether decreased or increased ER to mitochondria apposition is beneficial in AD models and at which stage of the pathology, together with each strategy’s influence on downstream pathological effects, would need to be set as a priority to progress in research on MERCS pharmaceutical modulators for neurodegeneration, especially AD.

Despite considerable advances in understanding many mitochondrial and MERCS processes in the pathogenesis of AD, precise mechanistic knowledge on MERCS physiology is lacking. Perhaps the best example of our lack of understanding of MERCS is the limited information we have on tethers and their role in different diseases. Although VAPB and PTPIP51 have been shown to be related to ALS (Stoica et al., 2014), such a direct connection has not been observed in AD. Indeed, a pressing issue in the field is to understand whether different tethers may be selectively affected in neurodegenerative disorders, generating a specific phenotypic “signature” of the disease. A limitation to current studies assessing proximity between selective pairs of tethers such as VAPB–PTPIP51 and IP3R–VDAC using the proximity ligation assay is that it could lead to misinterpretation of data; when a specific couple of tethers could be decreased, other tethering proteins could be upregulated leading to an overall increase in contact formation, which can only be assessed by structural apposition between the ER and mitochondria using split-green fluorescent protein (GFP)-based contact site sensors (SPLICS) or transmission electron microscopy (TEM). In fact, although VAPB levels and VAPB–PTPT51 proximity have been shown to be downregulated in sAD brains, we have recent data showing in *App* ^*NL-F*^ embryonic primary cortical neurons that while VAPB levels were decreased in these cells, overall MERCS structural apposition, measured through TEM, and functional apposition were upregulated (Dentoni et al., unpublished), hence hinting to several tethers playing a role in overall organelle juxtaposition in AD models.

Another concern that might arise from targeting specific molecules to ER–mitochondria tethers is that MERCS-related proteins are not exclusively located in these subcellular hubs of the cells. MERCS-enriched proteins are often also present in the bulk ER and mitochondria, playing a variety of physiological functions. Therefore, targeting a specific protein at MERCS might have off-target effects that need to be thoroughly investigated.

While tethers and regulators of tethering have received much attention in the MERCS field, methods altering the ER and mitochondria function through modulation of functional apposition between the two membranes should also be considered. Recently, we have shown that TOM70 depletion inhibits the IP3-linked ER to mitochondria Ca^2+^ transport independently from the interaction between the two membranes, in which MERCS structural characteristics remain unchanged upon TOM70 depletion (Filadi et al., 2018b). Indeed, TOM70 knockdown resulted in a decrease in co-localization between IP3Rs and mitochondria suggesting that TOM70 through its interaction with IP3Rs favors these channels’ functional recruitment close to mitochondria (Filadi et al., 2018b). Similarly, targeting IP3Rs and their interactors and stabilizers could prove an essential strategy to guarantee favorable ER to mitochondria apposition.

## 6 Conclusion

As our understanding of MERCS proteome and pathways are still incomplete, new methods aimed at systematically understanding the MERCS function may uncover novel avenues for therapeutic modulation in AD. Recently, methods to isolate, such as physical and biochemical characterization and detection of MERCS, with different reporters have been described elsewhere (Giamogante et al., 2020; Wilson and Metzakopian, 2021). These methods to identify (i.e., ContactID and SpliTurboID) and characterize MERCS morphology (i.e., EM and TEM), dynamics (i.e., FRET, PLA, and SPLICS), and function (i.e., cellular respirometry, quantification of ATP, Ca^2+^, Δψ_m_, ROS, and lipid levels) can be incorporated to pooled or arrayed high-throughput screenings to allow for novel recognition of drug candidates.

Although different open questions regarding the MERCS ultrastructure and composition remain to be further explored, we expect that as the local proteome at MERCS is deciphered and better models to study AD pathology emerge, we can develop small-molecule modulators to remodel the MERCS ultrastructure and function to restore the neuronal function in AD. We speculate future possibilities of using MERCS modulators in combination with disease-modifying treatments to decelerate or prevent disease progression even decades before clinical AD symptoms appear. In summary, these observations indicate that a complete understanding of pharmacodynamics and pharmacokinetic properties of MERCS modulators and refined mechanistic knowledge of MERCS-associated processes constitute instrumental processes for developing novel MERCS modulators for clinical therapeutic use in AD.

## Abbreviations

AD, Alzheimer’s disease; MERCS, mitochondria–endoplasmic reticulum contact sites; ER, endoplasmic reticulum; MAMs, mitochondria-associated endoplasmic reticulum membranes; OMM, outer mitochondrial membrane; IMM, inner mitochondrial membrane; PS, phosphatidylserine; PE, phosphatidylethanolamine; PC, phosphatidylcholine; Cer, ceramides.
